# Nursing brain drain from India

**DOI:** 10.1186/1478-4491-7-5

**Published:** 2009-02-02

**Authors:** Michael Hawkes, Mary Kolenko, Michelle Shockness, Krishna Diwaker

**Affiliations:** 1 Institute of Medical Sciences, University of Toronto, Toronto, Canada; 2 Faculty of Nursing, University of Toronto, Toronto, Canada; 3 Urban Promise, Toronto, Canada; 4 Department of Neonatology, Department of Pediatrics, Malankara Orthodox Syrian Church Medical College, Kolencherry, India

## Abstract

In response to recent findings regarding migration of health workers out of Africa, we provide data from a survey of Indian nurses suggesting that up to one fifth of the nursing labour force may be lost to wealthier countries through circular migration.

## Introduction

Migration of trained nurses from resource-poor countries to wealthier countries experiencing nursing shortages may exacerbate global health care inequities [1]. We wish to draw attention to the significant drain on India's nursing labour force due to "circular migration," using selected results from a recent survey.

## Discussion

We administered anonymous written questionnaires to a convenience sample of 99 nurses at a private hospital (Malankara Orthodox Syrian Church Medical College) in Kerala, India, in September 2007. Study participants had a median age of 27 years (range 22 to 57) and 96% were female. In terms of educational background, 98% of respondents had obtained a three-year nursing diploma while 2% had a four-year bachelor's degree in nursing. Of note, 20% of respondents had worked abroad, for a median of six years (range 2 to 15 years). Career time spent outside India accounted for 145 person-years (19%) out of a total of 621 person-years of total work experience reported in the survey. The receiving countries were: Oman (*n *= 4), Saudi Arabia (11) and Singapore (1). Working abroad was associated with older age (median 38 years versus 26 years, p < 0.001), more work experience (median 16 years versus four years, p < 0.001), and nursing seniority (44% of charge nurses versus 11% of staff nurses had worked abroad, p = 0.004).

With its high literacy rates and progressive education programmes, the state of Kerala trains a nursing workforce that is highly sought-after in the global labour market [2]. More than 50% of new nurse registrants in the United Kingdom in 2001 were from India, representing 0.21% of India's total nursing stock, and a comparable and increasing number of Indian nurses migrate to the United States of America annually [3]. The nursing workforce of Saudi Arabia, where most survey respondents had served a term abroad, is composed 83% to 95% of foreign nurses [4], many from South Asian countries [5].

Previous authors have noted that the Eastern Mediterranean is a common destination for the "circular migration" of Indian nurses, whereby nurses – motivated by higher salaries – work abroad temporarily, then return to their country of origin [6]. Consistent with this observation, older and more experienced nurses were more likely to have worked abroad in our cross-sectional survey, representing a significant group of nurses who had returned to India after a sojourn abroad.

It has been argued that circular migration does not produce the same degree of loss to a country's skilled labour force as permanent migration, since nurses ultimately return as a potentially enriched national resource [7]. However, our finding that an estimated 20% of nurses had worked abroad for a duration representing 19% of the cohort's pooled labour time suggests that temporary migration may have a profound and underestimated impact on the Indian nursing workforce. It should be noted that the added burden of permanent emigration could not be captured in this cross-sectional survey based on one hospital in India.

Previous studies corroborate our findings. A multi-centre survey of 448 practising nurses demonstrated that 63% of India's nurses intended to emigrate, citing dissatisfaction with working conditions and unhappiness with prevalent social attitudes toward nurses as motivating factors [6]. Another field study of nurses in Delhi indicated that the most common impetus for emigration was better income prospects overseas [8].

Our convenience sample was drawn from a private hospital, and consisted of predominantly Malayalam-speaking, Christian nurses, factors which have been associated with intention to migrate in a previous study of nurses in India [6], likely related to historical and cultural factors [9]. Nonetheless, intention to migrate among other linguistic and religious groups is also considerable: 50% among Sikhs and 48% among Hindus, in one report [6]. Of note, brain drain is not unique to the nursing or even health care professions: as many as 54% of India's top medical graduates leave the country [10], as well as large numbers of engineers and information technology professionals [8].

The positive economic, professional and social development that may result from circular nursing migration should be weighed against potential costs to supplier countries such as India. With a total health care workforce of 2.2 million and a population of over 1 billion inhabitants, the nursing density in India (7.9 nurses per 10 000 population) is well below international standards, and is inadequate to meet its current domestic health services needs [11]. With 769 public and private nursing education institutes across the country providing a three-year diploma programme (the educational background reported by the vast majority of nurses in our sample), India has the capacity to supply large numbers of English-speaking nurses [11], but should the double burden of training nurses for international export as well as filling local vacancies rest on this lower-income supplier country? Meanwhile, international agencies actively recruit nurses for work abroad and Kochi, the commercial centre of Kerala where our study was conducted, is one of three main recruiting hubs in the country [11].

## Conclusion

This snapshot of nursing "brain drain" in India suggests that up to one fifth of the nursing labour force may be lost to wealthier states through circular migration, as illustrated by one private hospital in southern India. As long as striking global disparities in nursing income persist, it will be difficult to stem the haemorrhage of nurses emigrating – albeit temporarily – in pursuit of better pay [4].

## Competing interests

The authors declare that they have no competing interests.

## Authors' contributions

MH conceived of the study, drafted the manuscript, and performed the statistical analysis. MK and MS participated in the design of the study. KD implemented and coordinated the study. All authors read and approved the final manuscript.
